# Lighting the Way: Anita Mahadevan-Jansen’s Journey Through Biophotonics and Beyond

**DOI:** 10.1117/1.BIOS.2.4.040501

**Published:** 2025-10-28

**Authors:** Darren Roblyer

**Affiliations:** Boston University, Boston, Massachusetts, United States

## Abstract

Anita Mahadevan-Jansen discusses her experiences of discovery and translation in biophotonics. The interview highlights her groundbreaking research as well as her impact as a mentor and leader in biomedical optics.

**Figure:**
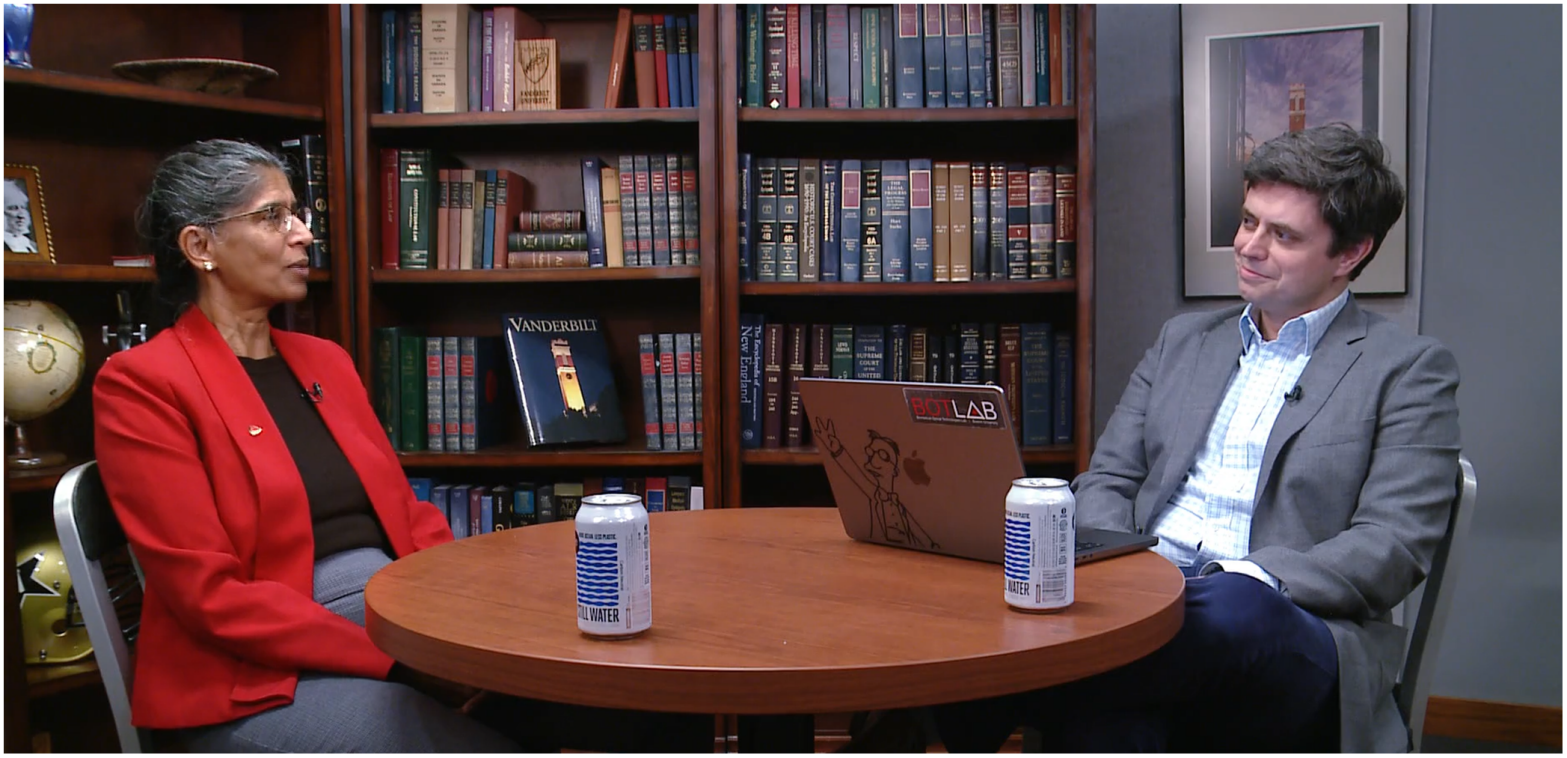
Anita Mahdevan-Jansen (left), Orrin H. Ingram Chair in Biomedical Engineering at Vanderbilt University, discusses translation and discovery in biophotonics with *Biophotonics Discovery* Editor-in-Chief Darren Roblyer (right). View a video recording of the interview at https://doi.org/10.1117/1.BIOS.2.4.040501.

In the world of biophotonics, few figures embody the spirit of innovation and mentorship like Dr. Anita Mahadevan-Jansen. From her early aspirations in Mumbai to her groundbreaking work at Vanderbilt University, Anita’s career is a testament to resilience, curiosity, and the transformative power of light.

## From Medicine to Physics to Biophotonics

Anita’s story begins in Bombay (now Mumbai), where she dreamed of becoming a doctor. Despite excelling in physics, chemistry, and biology, she missed medical school admission by a single point—a moment that would redirect her path toward physics and eventually biomedical engineering. Encouraged by her mentor, Dr. S.B. Patel, Anita pursued graduate studies in the U.S., overcoming obstacles like canceled GRE scores and navigating a new academic landscape.

Her first encounter with research in the U.S. was serendipitous. A chance meeting with Dr. Rebecca Richards-Kortum, then a new faculty member at UT Austin, led Anita to work on fluorescence and Raman spectroscopy for cervical cancer detection. That experience cemented her passion for translational research—applying physics to solve real-world medical problems: “I realized, okay, there’s a reason I didn’t go to medical school. That’s what I was meant to do—develop technologies to help populations of people instead of one patient at a time.”

## Reluctant Professor Turned Trailblazer

Anita never intended to become a professor. But when her husband, Dr. Duco Jansen, accepted a faculty position at Vanderbilt, Anita was offered a rare opportunity to start as a research faculty member. Initially hesitant about teaching, she struggled with classroom dynamics but eventually transformed her approach, embracing student-centered learning and becoming a respected educator.

Her persistence paid off. Anita secured her first NIH R01 grant for brain tumor demarcation using fluorescence, marking the beginning of a prolific academic career. Today, she holds the Orin H. Ingram Professorship of Biomedical Engineering and leads a vibrant research lab focused on surgical guidance, spectroscopy, neuromodulation, and open-access microscopy.

## A Discovery That Changed Surgery

Among Anita’s many contributions, her work on near-infrared autofluorescence (NIRAF) for parathyroid gland detection stands out. Sparked by a conversation with a surgical resident, the technology has revolutionized endocrine surgery by enabling real-time identification of parathyroid glands without dyes or contrast agents.

The journey from discovery to FDA clearance in 2018 involved rigorous testing, collaboration with industry, and global outreach. Today, NIRAF is used in clinical practice across the U.S., Europe, and Australia, with Anita continuing to educate surgeons on its proper use.

## Science Rooted in Humanity

Anita’s research is deeply personal. Her work on Raman spectroscopy for ear infection detection was inspired by her children’s struggles with otitis media. She believes in crafting projects around students’ passions and needs, fostering a lab culture of collaboration, critical thinking, and mutual respect.

She’s candid about being a demanding mentor but emphasizes the importance of independence and initiative. Her recruitment process is unique—students aren’t assigned to projects but co-create them, ensuring alignment with their interests and strengths.

## Mentorship and Lab Culture

Anita is known for her demanding but supportive mentorship style. “I am not an easy person to work for,” she says. “I expect my students to take initiative, come to me, not be scared to talk when they have issues.” Her lab operates as a community, with students involved in recruitment and project design. “I don’t recruit to a project. I say, ‘These are the kinds of stuff I do. If you want to work with me, let’s work together.’”

When asked who she most admires in the biophotonics field, Anita doesn’t hesitate: Bruce Tromberg. “He is such a kind human being, but also brilliant at the same time,” she says. “Everybody’s his friend—doesn’t matter what level they are. He’s someone I’ve always looked up to.”

## Championing Community and Critical Thinking

As a former president of SPIE (2022), Anita expanded her influence beyond biophotonics, connecting with global optics communities and advocating for diversity, accessibility, and student support. A founding senior advisor to *Biophotonics Discovery*, Anita believes the journal can unify and elevate the field: “I truly believe that the diversity of BIOS as a field—and this journal—can help.”

Passionate about fostering a culture of deep questioning in science, she encourages students and colleagues to challenge assumptions and think critically.

## Looking Ahead: Wine, Writing, and Retirement

With plans to retire in five years, Anita is already envisioning her next chapter. She dreams of becoming a sommelier, combining her love of wine with her scientific expertise—perhaps even using Raman spectroscopy to evaluate wine quality. She also hopes to explore creative writing, a passion nurtured through romance novels that help her unwind and stay inspired.

## A Legacy of Light

Anita Mahadevan-Jansen’s career is a masterclass in translational science, mentorship, and leadership. From developing life-changing technologies to shaping the next generation of researchers, she continues to illuminate the path forward—one photon at a time.

